# It Is Not Just a Matter of Motivation: The Role of Self-Control in Promoting Physical Activity in Older Adults—A Bayesian Mediation Model

**DOI:** 10.3390/healthcare12161663

**Published:** 2024-08-20

**Authors:** Nicola Camp, Roberto Vagnetti, Samuel Penner, Catarina Ramos, Kirsty Hunter, John Hough, Daniele Magistro

**Affiliations:** Department of Sport Science, School of Science and Technology, Nottingham Trent University, Nottingham NG11 8NS, UK; nicola.camp@ntu.ac.uk (N.C.); roberto.vagnetti@ntu.ac.uk (R.V.); samuel.penner2019@my.ntu.ac.uk (S.P.); catarina.fernandesramos@ntu.ac.uk (C.R.); kirsty.hunter@ntu.ac.uk (K.H.); john.hough@ntu.ac.uk (J.H.)

**Keywords:** older adults, physical activity, self-control, motivation, self-determination theory, mediation analysis

## Abstract

This study aimed to understand how psychological factors affect regular exercise in older adults, hypothesising that trait self-control mediates the relationship between motivation types (intrinsic, extrinsic, and amotivation) and exercise time. In this cross-sectional study, 430 older adults (mean age = 68.8 ± 6.72) completed questionnaires regarding their perceived trait self-control, motivation towards leisure activities, and level of physical activity. A Bayesian mediation analysis was performed, controlling for demographics. We documented positive direct (c′ = 0.021, 95%CI [0.001, 0.043]) and indirect (ab = 0.028, 95%CI [0.014, 0.043]) effects of intrinsic motivation on exercise, a fully mediated indirect effect of extrinsic motivation on exercise (ab = 0.027, 95%CI [0.011, 0.046]), and negative direct (c′ = −0.281, 95%CI [−0.368, −0.194]) and indirect (ab = −0.161, 95%CI [−0.221, −0.105]) effects of amotivation on exercise. There was no direct association between extrinsic motivation and exercise (c′ = 0.013, 95% CI [−0.013, 0.037]). In conclusion, trait self-control mediates motivation to influence exercise behaviour in older adults. Intrinsically motivated individuals resist sedentary living and show higher self-control, while extrinsically motivated ones rely on self-control and are more susceptible to non-adherence during mental fatigue. High amotivation is linked to less exercise and reduced self-control, suggesting potential non-compliance with structured exercise interventions.

## 1. Introduction

Older adults (individuals aged ≥65 years old) make up 18% of the total UK population at 11.9 million, a figure expected to increase to 24% by 2039 [1]. With ageing comes an increased reliance on health factors such as flexibility, balance, and strength to carry out daily activities, prevent injury, and remain independent [2]. Regular physical activity has been shown to maintain these attributes [3,4,5] and assists older adults in their ability to complete daily activities [6,7,8,9,10]. In addition, physical activity is known to reduce the risk of numerous health conditions that are prevalent among older adults, such as coronary heart disease, stroke, dementia, diabetes, arthritis, and osteoporosis, along with mental health conditions such as depression and anxiety [11,12,13,14,15,16,17]. Thus, it is no surprise that sedentary behaviour is a major cause of physical and mental health deterioration amongst the older population [18]. Given that 32% of UK adults aged 65–74 are classed as sedentary or ‘low activity’ [19] (National Health Service, 2019), it is essential that we identify further methods and initiatives to increase physical activity levels in the older population.

Previous exercise interventions aiming to increase exercise amongst older adults have displayed mixed results, particularly surrounding their capability to increase long-term adherence to exercise (>12 months; for reviews, see [20,21]). Whilst physical deterioration in cardiorespiratory fitness, balance, and muscle function are known to be barriers to exercise in the older population [22,23], it has been noted that psychological factors may influence older adults’ exercise behaviour to a similar degree [14,24,25]. Since one’s ability to adhere to exercise is influenced by multiple psychological constructs, if considered within interventions, they can enhance a shift in behaviour. Therefore, in order to increase older adults’ engagement in exercise, we must first identify the psychological constructs that are typically associated with a willingness to carry out exercise.

Motivation is one such construct that has featured in previous literature on the topic. Self-determination theory (SDT) posits that it is the type of motivation (rather than quantity) that is responsible for individual variation in behaviour [26]. Intrinsic motivation is regarded as the most ‘autonomous’ form of motivation, referring to the completion of an activity based solely on one’s own volition. Someone who is intrinsically motivated may carry out exercise due to its inherent satisfaction or to experience feelings of pleasure [27]. On the opposite side of the self-determination continuum, amotivation is considered a lack of the intention to act, which can be due to several reasons, for instance, the person does not feel competent, believes there is no connection between the action and the result, or does not have interest in the activity. Between amotivation and intrinsic motivation, extrinsic motivation is a form of motivation referring to engagement in an activity to obtain something that is not strictly related to the sole interest or pleasure in the activity itself. Extrinsically motivated behaviours are characterised by four types of regulations (external, introjected, identified, and integrated), corresponding to different levels of autonomous behaviour and internalisation processes [27]. Research has been consistent in detailing the beneficial effect of more autonomous forms of motivation and their capacity to incite behaviour change, particularly in the realm of physical activity [28,29]. Furthermore, it has been established that older adults who exercise habitually display higher levels of intrinsic motivation and identified regulation compared to short-term or non-exercisers [30,31].

Self-control (i.e., the inhibition of a dominant response, usually to delay short-term gratification for long-term reward) has also been widely associated with increased physical activity behaviour in older adults [14,32,33,34]. Originally, self-control was regarded as a limited resource, acting akin to a muscle that depletes upon repeated use or ‘exertion’ [35]. A plethora of studies employing a dual-task paradigm provided evidence for this hypothesis, with participants typically less able to exercise self-control in a second task after having ‘drained’ their self-control resources in a prior task [36,37]. Lately, however, this effect has been attributed to a motivational switch between ‘have to’ (extrinsic/controlled) and ‘want to’ (intrinsic/autonomous) motivation, which is said to occur in relation to self-control exertion [38,39]. Resultingly, there has been some recent speculation around the relationship between motivation and self-control. In the case of an older adult attempting to increase their level of physical activity, the completion of common, yet increasingly difficult day-to-day tasks may be draining enough to trigger a motivational switch from ‘have to’ to ‘want to’ motivation, which may result in a lack of adherence to exercise. Indeed, studies in the general population have demonstrated a combined influence of self-control and motivation on behaviour [40,41,42]. For example, Nurmi et al. [42] found that self-regulation partially mediates the positive relationship between autonomous motivation and exercise behaviour in adolescents. If interventions are able to promote exercise as a ‘want to’ activity (i.e., foster autonomous motivation) whilst simultaneously improving self-control strategies in older adults, more success may be achieved in long-term exercise implementation. However, it is still necessary to understand the relationships of these constructs in relation to the physical activity of older people and determine their magnitude.

Based on evidence from the literature, this study hypothesises that trait self-control mediates the relationship between motivation and physical activity level in older adults. Since self-control and autonomous motivation have shown positive relationships with health behaviour in older adults independently of each other, it would be useful to provide insight into the relationship between these psychological constructs and their combined influence on physical activity behaviour in older adults. These considerations may suggest successful intervention strategies to encourage increased physical and healthy activity in older adults. The aim of this study is to understand how psychological factors affect regular exercise in older adults, hypothesising that trait self-control mediates the relationship between types of motivation and time spent on physical activity.

## 2. Materials and Methods

### 2.1. Procedure

**Figure 1:**
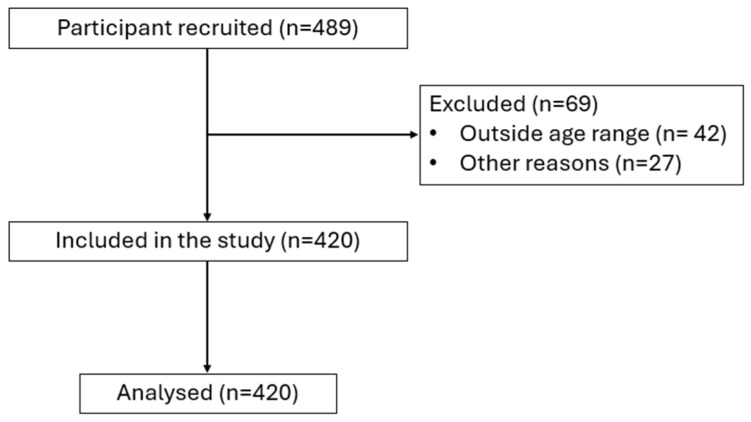
Flowchart of the study protocol.

This study featured a cross-sectional design, where participants completed self-report questionnaires at their own pace. Potential participants received emails with details of the questionnaire, the nature of the project, and a discretionary warning regarding sensitive topics featured in the questionnaire. Participants were given the option to complete the questionnaire via a weblink and asked to forward the questionnaire to other adults aged >60 years upon completion. Anonymity was maintained throughout data collection, and all participants gave written informed consent prior to completing the questionnaire. The study was approved by the Nottingham Trent University Ethics Committee (Case number: 19/20-08 V). The study protocol is in accordance with the ethical standards related to sports science [43]. A flowchart of the study protocol is presented in Figure 1.

### 2.2. Participants

Participants were recruited via convenience sampling from various parts of the United Kingdom. Adults over the age of 60 years were included in the study. Although older adults are usually classified as >65 years of age, it is not uncommon for studies to use samples of >50 years of age [30,31]. Our sample contained a total of 430 older adults (mean chronological age = 68.8, SD = 6.72). Almost one-third (32.3%, *n* = 139) of the participants were male, and 67.7% (*n* = 291) were female. Participants in this study were relatively well educated with 6.8% having completed tertiary education and 91% of participants having completed either secondary or tertiary education. Most participants (65.4%) were married at the time of data collection, with 25% either divorced or widowed. Only 34.2% of participants reported partaking in regular physical activity, and 75.8% of the sample were retired. Responses were collected from December 2021 to April 2022, after which data were analysed.

### 2.3. Measures

#### 2.3.1. Brief Self-Control Scale

Trait self-control (TSC) was measured using the Brief Self-Control Scale (BSCS) [44]. This instrument is a reduced 13-item form of the original 36-item scale, which has featured in previous research and shown good psychometric properties [36,44]. BCST measures people’s self-control by considering their ability to modify and override their inner responses and interrupt and refrain from undesired behaviours. Example items include “I am able to work towards long-term goals” or “I am good at resisting temptation”. Participants indicated how much each statement reflects them on a 5-point Likert scale, where 1= “Not at all” and 5 = “Very much”.

#### 2.3.2. Leisure Motivation Scale

Motivation towards leisure activities was assessed using the Leisure Motivation Scale (LMS) [45]. All items on the questionnaire are framed as answers to the question “why do you generally do your leisure activities”? The LMS assesses intrinsic motivation (e.g., “because my leisure activities give me a real high”); extrinsic motivation including identified, introjected, and external regulation (e.g., “because sometimes it allows me to be appreciated by others”); and amotivation (e.g., “I don’t really know; I don’t think that leisure activities suit me”). Participants answered each statement on a 7-point Likert scale. The LMS has shown high construct validity, discriminant validity, and high test–retest reliability [46].

#### 2.3.3. International Physical Activity Questionnaire

Participants’ physical activity behaviour was measured via retrospective self-reporting of their time spent in physical activity (TSPA) within the last 7 days using the International Physical Activity Questionnaire (IPAQ; [47]). Example questions include “during the last 7 days, how many days did you carry out moderate physical activity?” and “how much time did you usually spend exercising on one of those days”? The IPAQ includes questions covering four domains of physical activity (occupational activity, self-powered transportation, housework/gardening, and leisure time activity) and sedentary behaviours. For each dimension, the number of days per week and the amount of time spent daily on physical activity are documented. The IPAQ has shown high concurrent validity, construct validity, content validity, and test–retest reliability and has featured extensively in health literature [47,48]. Time spent doing physical activity was used for the analysis.

### 2.4. Statistical Analysis

**Figure 2:**
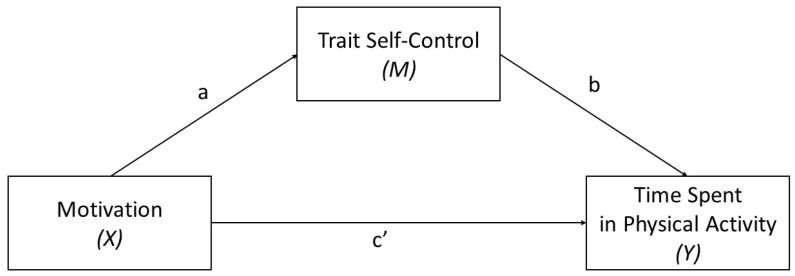
Mediation model for motivation, trait self-control (TSC), and time spent in physical activity (TSPA). a = effect of motivation on TSC, b = effect of TSC on time spent exercising, and c′ = direct effect of motivation on time spent exercising. Indirect effect of motivation on time spent exercising through TSC is calculated as the product of a and b. The total effect of motivation on time spent exercising (denoted c) is calculated as the sum of direct and indirect effects (c = c′ + ab).

According to the aim of the study, we performed a Bayesian mediation analysis to assess whether self-control mediates the relationship between motivation and physical activity. A simple mediation analysis explains the extent to which an exogenous variable (X) influences an endogenous variable (Y) through a mediator variable (M). Partial mediation occurs when X influences Y both directly and indirectly through a mediator M. Full mediation happens when X affects Y solely through the mediator M, with no direct effect. We built our models considering each type of motivation (i.e., intrinsic motivation, extrinsic motivation, and amotivation) as an exogenous variable (X), time spent exercising as an endogenous variable (Y), and trait self-control as the mediator (M); please refer to Figure 2. Simple regression analysis test is used for effects between exogenous variables and trait self-control (a), after which multiple regression analysis is used to test for direct effects of exogenous variables (c′) and trait self-control (b) on time spent exercising. The indirect effect of exogenous variables could be calculated as the product of paths a and b from the previous regression analysis (ab). Additionally, participants’ chronological age and sex (with females represented by the dummy variable ‘0’ and males by ‘1’) were considered as potential confounders. This was performed to correct the mediation models for potential bias arising from these demographic factors.

However, the sampling distribution of the mediated effect estimate may display non-normal characteristics, leading to potential bias in confidence interval estimates. To mitigate this issue, one method is to utilize Bayesian analysis for estimating the mediated effect. Unlike traditional approaches, Bayesian methods do not depend on the normality assumption of the sampling distribution of the estimates [49]. This is particularly advantageous in mediation analysis, as it avoids imposing restrictive normality assumptions, even when considering the indirect effects [49,50]. This method produces more reliable outcomes, especially with smaller sample sizes [51]. In contrast to conventional frequentist methods that hinge on maximum likelihood estimation, Bayesian approaches leverage prior information, adapting it through the incorporation of observed data to yield posterior distributions for the model parameters [52]. Using this approach, we derived posterior distributions for the parameters of the mediation models, including the posterior mean, standard error, standard deviation, and their corresponding 95% credible intervals (95%CIs). Credible intervals indicate that the true value of the parameter of interest is within the interval. These intervals were utilised to test if the coefficients of the model do not include zero with a 95% level of certainty. The posterior distribution was estimated using Markov Chain Monte Carlo (MCMC) sampling, with 500 burn-in observations and a tuning parameter set at 0.35. The algorithm was halted upon reaching convergence, determined by a Convergence Statistic [53] falling below 1.002. Convergence of the posterior distributions was also assessed visually through trace and autocorrelation plots. Additionally, the Posterior Predictive p (PPP) served as an indicator of the model’s adequacy, with a value of 0.50 considered an excellent fit and PPP < 0.10 serving as the threshold for model rejection [54]. A prior sensitivity analysis was performed comparing the estimated parameters utilising two different priors, a normal distribution, and a uniform distribution to assess the robustness of the results against the choice of the prior (Table S1, Supplementary Material).

A frequentist mediation analysis was also performed to compare the estimates of the two approaches; in this case, estimates were performed with maximum likelihood estimation (ML), direct effects were considered significant if they exhibited a *p*-value of <0.05, and the indirect effects were deemed significant if the lower and upper bounds of the bootstrapping confidence intervals did not contain 0. Confidence intervals were set at 95%, with bootstrapping of 5000 samples. The bootstrap approach is said to be the most suitable for smaller sample sizes due to the attenuation of type I error and is more reliable than other methods regardless of sample size for the same reason [55]. A sample size of at least 200 participants was considered adequate to achieve a power of 0.80, taking into account the presence of at least one path with a medium effect size [56]. Little’s test was conducted on the complete set of IPAQ, BSCS, and LMS questionnaires, revealing no significant differences (all ps > 0.05). This indicates that the data were likely MCAR. For cases where less than 5% of data points were missing, hot-deck imputation was used to fill in the missing values. Four participants had missing data for IPAQ, seven for BSCS, and eight for LMS. All data were analysed on IBM SPSS Statistics (Version 25) and AMOS (version 28.0).

## 3. Results

**Figure 3:**
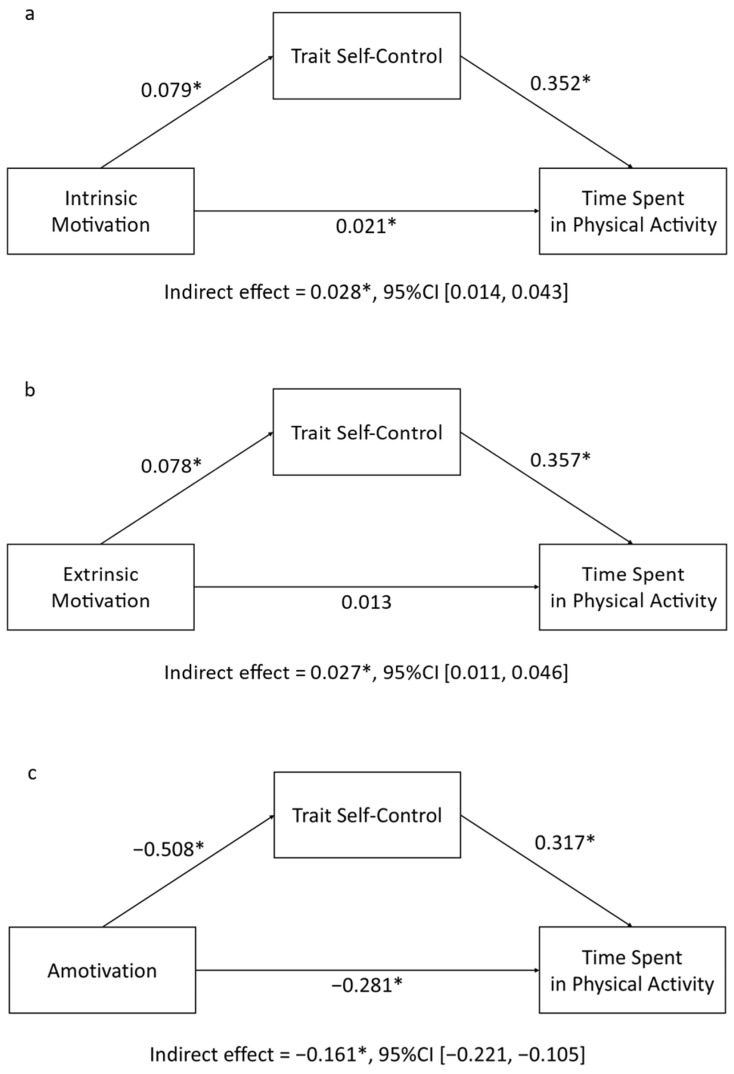
Schematic diagram of the Bayesian mediation analysis results. Trait self-control (TSC) significantly mediated the effect of (**a**) intrinsic motivation, (**b**) extrinsic motivation, and (**c**) amotivation on time spent in physical activity (TSPA). Note: * indicates a 95% credibility interval that does not contain zero.

Considering intrinsic motivation, the Bayesian mediation analysis indicated that it positively predicted trait self-control (a = 0.079, 95%CI [0.039, 0.118]). In turn, trait self-control positively predicted time spent exercising (b = 0.352, 95%CI [0.304, 0.404]). Intrinsic motivation was a predictor of time spent exercising (c′ = 0.021, 95%CI [0.001, 0.043]). The results indicate a mediation due to the indirect effect through the influence of intrinsic motivation of trait self-control (ab = 0.028, 95%CI [0.014, 0.043]) over a total effect of c = 0.048, 95%CI [0.024, 0.074]. Age resulted as a negative confounding variable for time spent in physical activity (mean estimate = −0.044, 95%CI [−0.148, −0.060]), while sex was associated with trait self-control (mean estimate = −2.27, 95%CI [−3.80, −0.695]). The model indicated an excellent fit (PPP = 0.50). The resulting model is depicted in Figure 3a.

Extrinsic motivation (Figure 3b) was positively associated with trait self-control (a = 0.078, 95% CI [0.030, 0.126]), which, in turn, influenced time spent exercising (b = 0.357, 95% CI [0.307, 0.408]). Interestingly, there was no direct effect of extrinsic motivation on time spent exercising (c′ = 0.013, 95% CI [−0.013, 0.037]), indicating that trait self-control fully mediated the relationship between extrinsic motivation and time spent exercising (ab = 0.027, 95% CI [0.011, 0.046]). The model demonstrated an excellent fit (PPP = 0.49). Age was associated with time spent exercising (mean estimate = −0.065, 95% CI [−0.120, −0.011]), while sex was associated with trait self-control (mean estimate = −2.38, 95% CI [−3.87, −0.848]).

Amotivation (Figure 3c) showed a negative association with trait self-control (a = −0.508, 95%CI [ −0.662, −0.345]), which was, in turn, associated with time spent exercising (b = 0.317, 95%CI [0.269, 0.366]). Amotivation itself was also a direct negative predictor of time spent exercising (c′ = −0.281, 95%CI [−0.368, −0.194]). Considering the total effect (c = −0.442, 95%CI [−0.540, −0.345]), there was an indirect effect between amotivation and time spent exercising, mediated by trait self-control (ab = −0.161, 95%CI [−0.221, −0.105]). The model demonstrated an excellent fit (PPP = 0.51). Age and sex were associated with time spent exercising (mean estimate = −0.062, 95% CI [−0.116, −0.009]) and self-control (mean estimate = −2.18, 95% CI [−3.64, −0.661]). A summary of the results is provided in Table 1 for better comparison.

The mediation analysis estimated by ML replicated these results. In all three models, age was significantly and negatively associated with time spent exercising, and sex was significantly associated with trait self-control (all ps < 0.05). The relationship between intrinsic motivation and time spent exercising (c′ = 0.021, *p* = 0.046) showed a significant indirect effect (ab = 0.028, 95% confidence intervals [0.012, 0.043]) through the relation with self-control (a = 0.079, *p* < 0.001), which, in turn, is associated with time spent exercising (b = 0.352, *p* < 0.001). The relationship between extrinsic motivation and time spent exercising was not significant (c′ = 0.013, *p* = 0.293), indicating full mediation due to the indirect effect (ab = 0.028, 95% confidence intervals [0.009, 0.047]) through its relationship with self-control (a = 0.078, *p* = 0.001), which, in turn, was significantly associated with time spent in physical activity (b = 0.357, *p* < 0.001). Amotivation was significantly associated with physical activity (c′ = −0.281, *p* < 0.001) and trait self-control (a = −0.505, *p* < 0.001), which, in turn, was associated with time spent exercising (b = 0.317, *p* < 0.001). The model indicated a significant indirect effect (ab = −0.160, 95% confidence intervals [−0.228, −0.092]).

## 4. Discussion

### 4.1. Relationships among Motivation, Trait Self-Control, and Exercise

Engaging in regular physical activity enables older adults to maintain their independence and lowers the risk of developing health problems [2,12,13]. However, adherence to intervention programs to increase physical activity is an area of concern which needs further consideration [20,21]. The literature suggests that motivational and self-control factors influence the success of healthy behaviour interventions such as exercise Therefore, understanding this relationship in older adults provides insights into the factors to be considered when promoting physical activity, which could potentially improve the outcomes of targeted interventions. The aim of this study was to examine the interrelationships among motivation, trait self-control, and physical activity behaviour in older adults.

Our results indicate that intrinsic motivation was significantly associated with time spent exercising, showing significant direct and indirect effects. Accordingly, older adults with higher levels of intrinsic motivation towards exercise (i.e., carry out exercise for personal enjoyment) tend to spend more time exercising, confirming the beneficial effect of autonomous motivation in the promotion of physical activity behaviour change [28]. Importantly, this relationship is partially mediated by trait self-control, whereby intrinsically motivated older adults display higher self-control skills which lead to stronger physical activity behaviour. These results support that of several previous studies which found self-control to mediate positive relationships between autonomous motivation and various behavioural outcomes, including subjective wellbeing [40,57], healthy eating behaviour [41], low athlete burnout [58,59], and exercise [42]. Consequently, self-control and intrinsic motivation are both important psychological factors influencing exercise behaviour in older adults.

Our results also indicate that trait self-control fully mediated the relationship between extrinsic motivation and time spent exercising, suggesting that when this form of motivation regulates physical activity, only their self-control skills (e.g., overriding inner responses) predict their behaviour. The observed lack of direct effect of extrinsic motivation on exercise behaviour is concurrent with the general literature [29] and with the self-determination theory [27], which states that extrinsic motivation is a less effective form of motivation in goal pursuit and goal attainment compared to autonomous motivation. The full mediation effect present in this study is also consistent with the results of Briki [40] who found that self-control fully mediated the relationship between extrinsic motivation towards physical activity and subjective wellbeing. Likewise, Milyavskaya et al. [41] demonstrated a full mediation of barrier perception/effort spent overcoming barriers (i.e., self-control exertion) in the relationship between ‘have to’ controlled motivation and low goal attainment.

The observed partial mediation effect for intrinsic motivation, trait self-control, and exercise alongside a full mediation effect for extrinsic motivation, trait self-control, and exercise may suggest that, in times of mental fatigue or ego depletion, autonomously motivated older adults are able to count on intrinsic motives to carry out physical activity. As evidenced by the lack of a direct effect between extrinsic motivation and exercise, older adults may not be able to rely on extrinsic motives during times of mental fatigue, instead relying fully on self-control to carry out physical activity, which could be more likely to fail [38,60,61]. If self-control is a limited resource, then older adults, especially those who display ‘have to’ motivation towards exercise and experience a higher occurrence of daily control-draining activities, may require ‘heightened’ self-control to carry out physical activity in the face of distraction. Likewise, if prior self-control exertion results in a shift in ‘have to’ to ‘want to’ motivation (i.e., controlled to autonomous) [38,62], then it would follow that older adults who display intrinsic motivation towards exercise are able to withstand the motivational switch caused by increasingly resource-draining day-to-day tasks [8], whereas those who display extrinsic motivation towards exercise are more susceptible to non-adherence, choosing another ‘want to’ activity (e.g., knitting) instead of exercise. Older adults who are extrinsically motivated are fully reliant on self-control to maintain exercise which would fit into the process model of self-control [38]. As evidenced by Milyavskaya et al. [41], these individuals perceive more barriers to exercise, therefore relying heavily on self-control to overcome these obstacles as opposed to personal incentives, intrinsic values, and enjoyment. This would explain why trait self-control fully mediated the relationship between extrinsic motivation and exercise in the present study, as these individuals fully rely on self-control to resist the motivational switch from ‘have to’ to ‘want to’, whereas intrinsically motivated individuals already ‘want’ to exercise and, subsequently, only partially rely on self-control when their motivation inevitably shifts. We should emphasise that our results also indicate that higher levels of extrinsic motivation can foster the self-control resources of older people; however, since executive functions are widely known to decline with age [61,63,64], it seems unlikely that the use of extrinsic motivators during exercise interventions would be successful as it would induce a greater reliance on already-declining self-control.

Regarding amotivation, mediation analysis indicated a direct and indirect negative effect on exercise behaviour, whereby older adults who display a lack of motivation towards exercise also exhibit lower self-control skills, both of which constitute weaker exercise behaviour. Our results partly support that of the wider literature; in a review of 13 studies testing amotivation and physical activity in the general population, 9 of the studies found a negative association between amotivation and physical activity, whereas the other 4 studies found no association [29]. In terms of the older adult population, only one previous study has directly measured the relationship between amotivation and physical activity; it also found a significant negative association [65]. However, other research has also shown that a large proportion of non-adhering older adults cite lack of value or perceived benefit of physical activity as a reason for non-adherence (e.g., “I feel the same whether I exercise or not”, ”I am not interested in exercise”) [66,67], an aspect which is typically considered among the main reasons associated with amotivation [68]. The link between amotivation and self-control has scarcely been studied. Negative relationships between amotivation and self-control have been found in problem gamblers and problem gamers [69,70]. Likewise, amotivation has been shown to predict negative behaviour (i.e., athlete burnout) indirectly through its effect on self-control [59], mirroring the present results. Regarding older adults, this study is the first to provide evidence of a significant negative correlation between amotivation and self-control skills. The mediating role of self-control also supports the use of interventions targeting this skill (e.g., [71]) in the older adult population to improve physical activity. However, it suggests certain limitations in the presence of high levels of amotivation. The relationship suggests that the more unmotivated older adults are performing physical activity (e.g., they do not care about the activity or do not perceive beneficial outcomes), the fewer self-monitoring resources they “invest” in this task, while they are likely to be more invested in other “have to” activities. These findings are meaningful since amotivation is a typical symptom of depression [72,73,74], which affects 28.4% of the older adult population globally [75]. Additionally, since depression is known to be detrimental to self-control and cognitive function by extension, especially in older adults [76,77,78], the significant negative association between amotivation and trait self-control found in the present study suggest that further research should consider the role of mental illness in the relationship between motivation and self-control in older adults.

Finally, it is worth mentioning that sex is associated with trait self-control, suggesting that women could have an advantage in this skill, in agreement with other evidence in the literature [79,80]. Age was linked to the duration of time spent on physical activities, showing that as individuals age, their exercise time generally diminishes. This is reflective of the natural decline in physical abilities that occurs with ageing, which is essential for daily activities [7,81].

### 4.2. Practical Considerations and Future Directions

Self-control resources translate into strategies and efforts to engage in physical activity and are closely related to motivation [42]. Considering the self-determination theory framework [26,27], partial mediation by intrinsic motivation reflects the inherent satisfaction derived from physical activity, which promotes further engagement and investment in self-control resources. In contrast, extrinsic motivation, where internalisation processes are still developing and the activity is not pursued for pleasure or interest, does not have the same effect and leads to full mediation. Consequently, older adults may be motivated to engage in physical activity for various reasons, whether intrinsic or extrinsic, and will invest their resources accordingly. Conversely, amotivation is associated with a lack of intention to engage in physical activity and a reduced investment in related resources, as indicated by the negative associations identified.

Interventions to increase physical activity in older adults should aim to foster an intrinsic, autonomy-supportive climate through the provision of ample choice for activity (type, duration, and solo/group), sufficient information to aid participants in selecting an activity, constant feedback, and individualised goal setting [82,83,84]. Existing interventions typically do not provide any choice of activity which may be ignoring the psychological needs of participants and, thus, reduce the likelihood of long-term exercise adherence [85]. Indeed, intrinsic motives and autonomy have been shown to increase the likelihood of long-term, sustained exercise behaviour amongst older adults [30]. The prospect of increasing exercise participation amongst unmotivated older adults is challenging as there is a low likelihood of recruitment into any exercise intervention, nor is it likely that this group will start exercising habitually without any form of intervention. Multidisciplinary interventions should be recommended, for instance, the inclusion of motivational interviewing, a technique proposed for use with lower-motivated and unmotivated individuals to improve engagement in health behaviours [86], and can result in promising health behavioural change in older adults, such as physical activity [87].

Since amotivation affects physical activity negatively through self-control, low adherence rates in a structured physical activity intervention to increase exercise amongst unmotivated older adults would be common. Future research might find success, therefore, by integrating physical activity with other hobbies such as gardening, attending places of worship, or spectating sports. For example, a ‘walk-to-stadium’ scheme could be used for older adults who live locally to a sports club, in which walking activity could be added up and exchanged for sports tickets. The integration of physical activity with other hobbies (of the participant’s choice) could reduce both perceived obstacles and a lack of motivation as other hobbies cultivate autonomous motivation, therefore making them resistant to lapses in self-control [41,88].

### 4.3. Limitations

Despite the interesting results, our study has some limitations. The variation in ageing effects experienced within the 60–85 age group should be acknowledged. In this regard, the present study considered participants’ age as a confounder to control for age-related bias. Additionally, since the psychological characteristics of self-control and intrinsic motivation are socially desirable and generally deemed as ‘good’ traits, a response bias could have occurred in our results [89]. The possibility of sampling bias should also be noted; recipients of the questionnaire e-mail who enjoy physical activity or exhibit an interest in psychology/science would be more likely to complete the survey. This study relied on self-reports to obtain data on psychological variables and physical activity, which may introduce bias and represent a limitation.

## 5. Conclusions

The present study aimed to provide insight into the psychological determinants of exercise adherence in the older population. We demonstrated that trait self-control mediates the relationship between various types of motivation and the amount of exercise completed within a prior 7-day period. Older adults with intrinsic motivation towards exercise are more able to withstand the non-adherence to physical activity caused by increasingly resource-draining day-to-day tasks. When extrinsic motivation regulates the physical activity of older adults, only their self-control skills support the implementation of physical activity. Additionally, trait self-control partially mediated the negative relationship between amotivation and physical activity. This study is the first to demonstrate such relationships. This study further confirms the extensive literature indicating that motivation can drive behaviour change in physical activity. However, it highlights the crucial role of the type of motivation involved. Additionally, older adults’ self-control resources, which support the inhibition of dominant responses for long-term rewards, have emerged as a mediating factor in these dynamics. Accordingly, assessing older adults’ motivation and self-control resources to tailor specific interventions could result in better physical activity adherence and well-being for this population. The assessment and involvement of these psychological resources represent a significant turning point in supporting older adults to engage in physical activity. Future studies should further consider these psychological variables when designing intervention strategies. For instance, for individuals with high extrinsic motivation and amotivation to perform physical activity, self-control is the primary factor that can support their engagement. Enhancing self-control resources in such situations may lead to improved benefits. Additionally, this strategy should be combined with leveraging intrinsic motivation by designing ‘want to’ physical activity contexts.

These results suggest that motivation and self-control are conducive to exercise behaviour in older adults and should be considered in future interventions that aim to increase physical activity amongst the older population, with particular emphasis on methods to combat amotivation towards exercise. With reference to our results, future theoretical research should aim to develop our understanding around the relationship among motivation, self-control, and behaviour. This understanding will underpin future methods for inciting behaviour change.

## Figures and Tables

**Table 1:** Estimates [95% CIs] of the Bayesian mediation analysis results between types of motivation and trait self-control (TSC) on time spent in physical activity.

Type of Motivation	Effect of Motivation on TSC (a)	Effect of TSC on TSPA (b)	Direct Effect of Motivation on TSPA (c′)	Total Effect (c)	Indirect Effect (ab)
Intrinsic Motivation	0.079 * [0.039, 0.118]	0.352 * [0.304, 0.404]	0.021 * [0.001, 0.043]	0.048 * [0.024, 0.074]	0.028 * [0.014, 0.043]
Extrinsic Motivation	0.078 * [0.030, 0.126]	0.357 * [0.307, 0.408]	0.013 [−0.013, 0.037])	0.041 * [0.006, 0.072]	0.027 * [0.011, 0.046]
Amotivation	−0.508 * [−0.662, −0.345]	0.317 * [0.269, 0.366]	−0.281 * [−0.368, −0.194]	−0.442 * [−0.540, −0.345]	−0.161 * [−0.221, −0.105]

* Indicates a 95% credibility interval that does not contain zero.

## Data Availability

The datasets used in this study are available from the corresponding author on reasonable request.

## Supplementary Materials

The following supporting information can be downloaded at: https://www.mdpi.com/article/10.3390/healthcare12161663/s1, Table S1: Sensitivity analysis due to prior selection.
