# Supplementary material for: It Is Not Just a Matter of Motivation: The Role of Self-Control in Promoting Physical Activity in Older Adults—A Bayesian Mediation Model
Source: Healthcare (Basel). 2024 Aug 20;12(16):1663. doi: 10.3390/healthcare12161663 (PMC11353447; doi:10.3390/healthcare12161663)
Supplement: Supplementary file 1 [file healthcare-12-01663-s001.zip › healthcare-3123151-supplementary.pdf]

Table S1 Sensitivity analysis due to prior selection.

	Intrinsic Motivation				Extrinsic Motivation				Amotivation			
	Prior I		Prior II		Prior I		Prior II		Prior I		Prior II	
	Estimate	SE	Estimate	SE	Estimate	SE	Estimate	SE	Estimate	SE	Estimate	SE
Motivation Component -> Self-Control	0.079	0.001	0.078	0.001	0.078	0.001	0.078	0.001	-0.508	0.002	-0.502	0.002
Self-Control -> Time Spent in PA	0.352	0.001	0.351	0.001	0.357	0.001	0.357	0.001	0.317	0.001	0.317	0.001
Motivation Component -> Time Spent in PA	0.021	0.001	0.021	0.001	0.013	0.001	0.013	0.001	-0.281	0.001	-0.283	0.001
Indirect effect	0.028	0.001	0.027	0.001	0.026	0.001	0.027	0.001	-0.161	0.001	-0.159	0.001
Confounders												
Gender -> Self-Control	-2.279	0.020	-2.300	0.018	-2.368	0.021	-2.324	0.022	-2.182	0.020	-2.186	0.023
Gender -> Time Spent in PA	-0.128	0.013	-0.134	0.013	-0.114	0.008	-0.131	0.009	-0.200	0.011	-0.200	0.012
Age -> Self-Control	-0.044	0.001	-0.045	0.001	-0.037	0.001	-0.036	0.001	-0.033	0.001	-0.035	0.001
Age -> Time Spent in PA	-0.067	0.001	-0.068	0.001	-0.065	0.001	-0.067	0.001	-0.062	0.001	-0.061	0.001
